# Experiences and perceptions of continuous deep sedation: An interview study among Dutch patients and relatives

**DOI:** 10.1111/hex.13869

**Published:** 2023-10-11

**Authors:** Louise Annemoon Jonker, Madelon T. Heijltjes, Judith A. C. Rietjens, Agnes van der Heide, Geeske Hendriksen, Johannes J. M. van Delden, Ghislaine J. M. W. van Thiel

**Affiliations:** ^1^ Department of Bioethics and Health Humanities, Julius Center for Health Sciences and Primary Care University Medical Center Utrecht Utrecht The Netherlands; ^2^ Department of Pediatrics Diakonessenhuis Utrecht Utrecht The Netherlands; ^3^ Department of Public Health, Erasmus Medical Center Erasmus University Rotterdam The Netherlands; ^4^ Department of Design, Organization and Strategy, Faculty of Industrial Design Engineering Delft University of Technology Delft The Netherlands

**Keywords:** continuous deep sedation, end of life, family, palliative sedation, patient, qualitative research, relative

## Abstract

**Background:**

The incidence of continuous deep sedation (CDS) has more than doubled over the last decade in The Netherlands, while reasons for this increase are not fully understood. Patients and relatives have an essential role in deciding on CDS. We hypothesize that the increase in CDS practice is related to the changing role of patients and relatives in deciding on CDS.

**Objective:**

To describe perceptions and experiences of patients and relatives with regard to CDS. This insight may help professionals and policymakers to better understand and respond to the evolving practice of CDS.

**Methods:**

Qualitative interviews were held with patients and relatives who had either personal experience with CDS as a relative or had contemplated CDS for themselves.

**Results:**

The vast majority of respondents appreciated CDS as a palliative care option, and none of the respondents reported (moral) objections to CDS. The majority of respondents prioritized avoiding suffering at the end of life. The patients and families generally considered CDS a palliative care option for which they can choose. Likewise, according to our respondents, the decision to start CDS was made by them, instead of the physician. Negative experiences with CDS care were mostly related to loss of sense of agency, due to insufficient communication or information provision by healthcare professionals. Lack of continuity of care was also a source of distress. We observed a variety in the respondents' understanding of the distinction between CDS and other end‐of‐life care decisions, including euthanasia. Some perceived CDS as hastening death.

**Conclusion:**

The traditional view of CDS as a last resort option for a physician to relieve a patient's suffering at the end of life is not explicit among patients and relatives. Instead, our results show that they perceive CDS as a regular palliative care option. Along with this normalization of CDS, patients and relatives claim a substantial say in the decision‐making and are mainly motivated by a wish to avoid suffering and exercise control at the end of life. These distinct views on CDS of patients, their relatives and healthcare providers should be reconciled in guidelines and protocols for CDS.

**Patient or Public Contribution:**

One of the authors in our team (G. H.) has experience with CDS as a relative and ensured that the patient/relative viewpoint was adequately reflected in the design and conduct of our study. In the preliminary phase of our study, G. H. adjusted the topic list so it was better adapted to the current practice of CDS. During the data analysis, G. H. read several interviews and took part in the open and critical discussion on central themes and core concepts as an important member of the author team, thereby guaranteeing the central position of the patient/relative perspective in our final research outcome.

## 1 INTRODUCTION

Continuous deep sedation (CDS) is a form of palliative sedation that relieves suffering at the end‐stage of life by continuously lowering the consciousness of the terminally ill patient until death.^1^ According to the Dutch guideline—of which core elements are presented in Table 1—the indication for CDS is the presence of refractory symptoms causing intolerable suffering in the last weeks or days of life. Symptoms are deemed refractory when they cannot be controlled to an acceptable degree within a reasonable time or without unacceptable side effects.^2^, ^3^, ^4^ Classic examples of refractory symptoms are severe dyspnea, pain and delirium.^5^ It is internationally viewed primarily as a last resort medical decision, and the patient cannot opt for CDS unless the preconditions are fulfilled in the opinion of the physician.^2^, ^3^, ^4^, ^6^

**Table 1:** Core elements of the 2009 version of the RDMA guideline on the use of CDS.^a^

Continuous sedation is the practice of intentionally lowering the consciousness of patients continually until death at the end stage of life to reduce unbearable suffering. Continuous sedation is always administered in the final stage of life. The patients concerned are dying and experiencing unbearable suffering. Medical indications are present when one or more intractable or ‘refractory’ symptoms are causing the patient unbearable suffering. A symptom is considered to be refractory if none of the conventional modes of treatment is effective or fast‐acting enough, and/or if these modes of treatment are accompanied by unacceptable side effects. A precondition for the use of continuous sedation is the expectation that death will ensue in the reasonably near future—that is, within 1–2 weeks. Next to physical suffering, existential suffering can also play a role in determining if suffering is unbearable and refractory. However, existential suffering alone cannot be an indication to start continuous sedation. When patients suffer from existential problems, it is recommended to consult an expert in psychosocial and spiritual care. Palliative sedation is a medical response to a serious medical problem. A patient cannot opt for continuous sedation unless the indications and preconditions for this option are fulfilled. Only if the indications are present, in the physician's opinion, and the preconditions have been met does continuous sedation become a right that the patient may choose to exercise. The general rule is that palliative sedation should not be initiated without the consent either of the patient himself or, if he is decisionally incompetent, his representative. The patient's condition may make it necessary to administer acute sedation. This means sedating a patient in a situation in which a complication (frequently one, i.e., life‐threatening) suddenly occurs that causes unbearable suffering. In that case, the physician may decide that acute sedation is the only sound option for alleviating the patient's suffering at the point in time. Where a physician has doubts regarding his own expertise or has difficulty balancing the different considerations involved in deciding whether to start CDS, it is standard professional practice to consult the appropriate expert in good time. Midazolam is the drug of choice, the use of morphine as a sedative is bad practice. In principle, there is no artificial administration of fluids during the provision of continuous sedation. Continuous deep sedation differs from euthanasia in that its aim is not to shorten life.

Abbreviations: CDS, continuous deep sedation; RDMA, Royal Dutch Medical Association.

^a^ The 2009 version of the RDMA guideline was the actual version during the time of the interviews.

In recent years, the practice of CDS in The Netherlands has expanded significantly from 8.2% of all deaths in 2005 to 18.3% in 2015. This increase was observed in all age groups and for all causes of death. However, the increase was most prominent in patients over 80 years of age and patients dying from cancer or cardiovascular disease.^7^ CDS is a far‐reaching intervention and many have argued that it can only be justified on serious and proportionate grounds.^8^, ^9^, ^10^, ^11^ The increase in its use calls for a profound understanding of current practice.

European research on CDS has mainly focused on the perceptions of healthcare providers (HCPs),^12^, ^13^, ^14^, ^15^, ^16^, ^17^ whereas the experience of patients and relatives has received less attention. Their role in the decision‐making on end‐of‐life care has, however, been recognized as indispensable.^18^ Indeed, over the last decade research shows an increasing concern of HCPs for the wishes of patients and relatives with respect to CDS, and patients desire a more active role in end‐of‐life decisions.^16^, ^17^, ^19^ This stands in contrast with the ‘last resort’ view of CDS in which its indication is solely a medical one and the decision about its use should be made by the physician. The rise in the frequency of CDS could be associated with a change in the role of the patient in decision‐making. Better insight into the views and experiences of patients and relatives may contribute to the understanding of the increase in the use of CDS in The Netherlands and may help professionals and policymakers to adequately respond to the evolving practice of CDS.

## 2 METHODS

### 2.1 Design

We conducted a qualitative study using semistructured interviews. The interviews were guided by a topic list based on CDS literature^20^, ^21^, ^22^, ^23^, ^24^ and input by author G. H. who experienced CDS as a relative and provided us with a detailed description of her experience on current CDS practice. The topic list was tested during three pilot interviews and adjustments were made accordingly in discussion with G. H. An English version of the topic list can be found in Supporting Information: 1. All respondents were questioned on their individual views of CDS and, if applicable, on their experience of CDS as a relative.

In our study, CDS was defined according to the definition of the Royal Dutch Medical Association (Table 1). However, respondents may not always be aware of the exact definition of CDS. To ensure respondents were discussing CDS and not another palliative care intervention, they were questioned on their understanding of the concept of CDS. In case a respondent understood CDS in ways contrary to the general definition of CDS, this was corrected during the interview using teach‐back.

### 2.2 Study sample

We recruited a sample from an existing panel of laypersons at the University Medical Center Utrecht, The Netherlands (UMCU). This panel consisted of patients who received care at the UMCU and indicated their willingness to partake in scientific research. Additional respondents were recruited through the personal network of the researchers. Potential respondents were included if they had experienced CDS with a close relative or had contemplated CDS for themselves. Respondents participating as relatives could be a partner, family member or friend of a person who had received CDS, but not someone who took care of the patient professionally. The potential respondents were invited by email and people who expressed their interest in participation received further information, after which they were asked to give informed consent for use of their data for the purposes of this research.

### 2.3 Data collection

The interviews were conducted by L. A. Jonker, at that time a senior medical student, and M. T. Heijltjes, a physician working as a PhD student, who was trained in qualitative research. L. A. Jonker was supervised by M. T. Heijltjes and G. J. M. W. van Thiel, an experienced qualitative researcher. The interviews were held between November 2019 and June 2021. The interviews took place at a location suitable to the respondent, but from March 2020 onwards interviews were exclusively conducted through telephone or an online video connection, due to official regulations related to the COVID‐19 pandemic. The inclusion of respondents continued until the research group concluded that conceptual saturation was reached.

### 2.4 Data analysis

We conducted a thematic analysis of the data that was partly deductive and partly inductive in nature. Experiences with cases were analyzed when they had occurred after the—at the time—most recent guideline on CDS by the RMDA (2009). We excluded reports of intermittent sedation and a case in which the respondent was involved in a professional role.

The data analysis consisted of four phases; as a first step, L. A. Jonker read and reread all transcripts thoroughly. Subsequently, L. A. Jonker coded all transcripts in light of the research aim using NVivo software version 12.6. Additionally, G. J. M. W. van Thiel and M. T. Heijltjes individually read and coded four transcripts. The coding was then discussed and refined. In the third phase, the codes were categorized and bundled into overarching concepts, to create an overview of the results. Lastly, using several open and critical conversations with all authors, central themes and core categories were identified with the main purpose of answering the research question. Illustrative quotes were translated from Dutch into English.

### 2.5 Ethics approval and reporting

The medical research ethics committee METC Utrecht confirmed that under Dutch Law, this research is exempt from review by a medical research ethics committee (protocol number 19‐435/C). This study is reported according to the COmprehensive consolidated criteria for REporting Qualitative research.^25^

## 3 RESULTS

**Figure 1:**
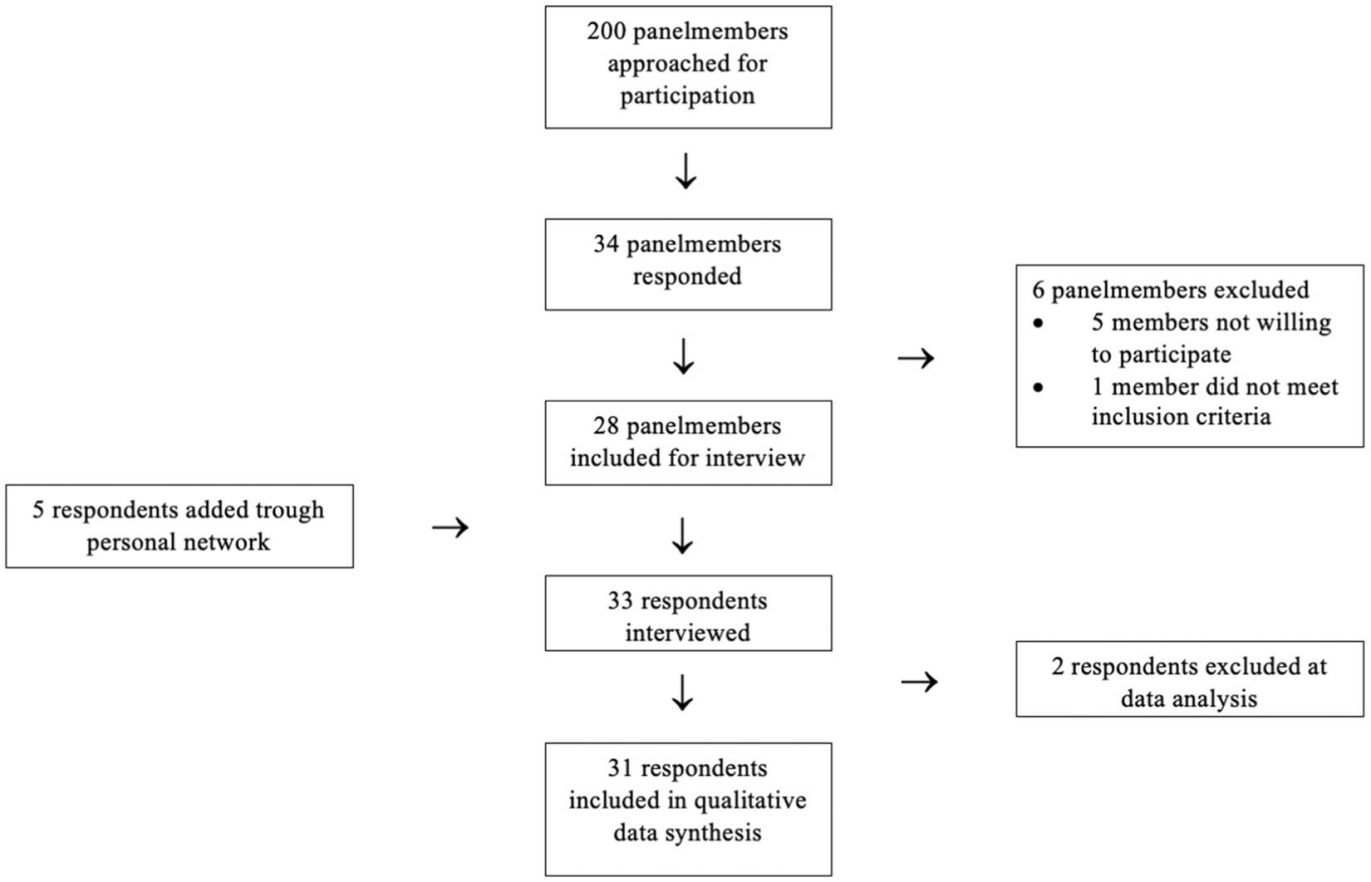
Inclusion diagram.

Two‐hundred members of the patient panel were invited to participate. In total, 34 panel members responded of which six were excluded. Five additional respondents were added through personal network. In total, 33 respondents were interviewed. During data analysis two respondents were excluded as we were not able to determine with certainty that the interviews were about CDS: these respondents did not demonstrate adequate understanding of CDS and did not receive a teach‐back from the interviewer. All of the 31 remaining respondents displayed a correct understanding of CDS either by their own knowledge or after the teach‐back provision (Figure 1).

All respondents recruited via the UMCU patient panel (26) were included as patients, as they all received care for a variety of serious illnesses at the UMCU and therefore in a situation in which they had contemplated or discussed the option of CDS. Twenty‐six respondents had experience as a relative of a patient for whom CDS was considered (5) or performed (31) and some respondents reported on more than one CDS case. Characteristics of the respondents and of the cases are listed in Table 2. The majority of the discussed CDS cases dated back less than five years. The duration of the interviews was between 30 and 90 min. We identified six themes relevant to our research question.

**Table 2:** Respondent and case characteristics.

*N* (%)	
*Respondent characteristics* (*n = 31*)	
Age	
40–49	2 (6)
50–59	6 (19)
60–69	15 (48)
70–79	8 (26)
Gender	
Female	19 (61)
Male	12 (39)
Level of education^a^	
Higher	25 (81)
Lower	6 (19)
Religion	
None	20 (65)
Christian	9 (29)
Unspecified	1 (3)
Buddhist	1 (3)
Contemplated CDS as a patient	
Yes	26 (84)
No	5 (16)
Experience with CDS as a relative	
Yes	26 (84)
No	5 (16)
Case characteristics (*n* = 36^b^)	
CDS was provided	
Yes	31 (86)
No^c^	5 (14)
Medication used to achieve CDS according to respondent	
Midazolam	17 (55)
Morphine	6 (19)
Unclear	8 (26)
Respondent present during CDS care provision	
Yes	31 (86)
No	5 (14)

Abbreviations: CDS, continuous deep sedation; HCP, healthcare providers.

^a^ Level of education was defined according to the International Standard Classification of Education 2011: higher education included all individuals who had a university degree (bachelor, master or doctoral) and lower education included all individuals who had either no education, primary education alone, secondary education alone or postsecondary nontertiary education.

^b^ Some respondents discussed more than one CDS case.

^c^ In these cases CDS was discussed by the family with an HCP, but eventually, CDS was not provided due to a variety of reasons.

### 3.1 Reasons for starting CDS

None of the respondents reported (ethical) objections to CDS. All respondents indicated the importance of a peaceful and painless deathbed. Suffering was considered unacceptable by most respondents and was the main reason for starting CDS in the discussed cases. Pain was the major source of intolerable suffering, followed by delirium, dyspnea and nausea. Existential suffering, due to fear, loss of identity, and a sense of pointlessness, was also considered unacceptable suffering and a motivation to start CDS in several cases.

Interviewer: What made her so uncomfortable?

Respondent: Well, I think a sort of fear of death. I think not knowing what will happen, and how long it will take.

Sometimes the relative asked to reduce suffering, which led to the decision of an HCP to initiate CDS:

Respondent: Well, after we specifically asked for something to calm her down, the health care workers decided to give her a butterfly needle which was used to administer morphine and midazolam.

When discussing their own death, several respondents brought up that they would consider CDS for themselves to reduce the suffering of their relatives as a consequence of their own suffering. Other respondents mentioned the wish for CDS in case they would become severely dependent on care. For example, when admission to a nursing home is inevitable, or when there is a necessity for life supporting measures such as mechanical ventilation.

### 3.2 The decision‐making process towards CDS

The respondents in our study generally believed that the decision for CDS was made by the patient, and not the physician. They regarded starting CDS as a matter of choice between other end‐of‐life care options, such as euthanasia. Physicians were valued as advisors, and guided the decision‐making process but were not seen as the one making the final decision to start CDS.

Respondent: Yes, we discussed this with him, the doctor and me. I mean, he [the patient] had to make a decision, but we discussed it together at home.

In case the patient was cognitively impaired, relatives made the decision together with the physician. In a few cases, the respondent reported that the physician initiated CDS without involving relatives in the decision. This was mostly experienced as frustrating by the relatives.

Respondent: At a certain moment it [CDS] was started, and then my youngest sister became very angry because it wasn't discussed with us as family. She said: this can't just be a statement [starting CDS], I want to discuss this with the treating physician!

Several respondents had asked an HCP involved in the care of their relative for measures to reduce the patient's suffering and some had explicitly asked for CDS, which was subsequently granted by the attending physician. Incidentally, relatives or HCPs convinced patients to start with CDS, as they thought that the suffering had become too intense.

Respondent: But eventually the doctor, together with her [the patient's] husband, kind of convinced her. She of course knew that things were ending. I think eventually she also felt, well, very tired. But, and I'm not saying it was against her will because then the doctor wouldn't do it of course, but they had to convince her.

The vast majority of respondents indicated that they wanted to make the decision to start CDS for themselves, in case they would need it in the future. If this were impossible, for example, due to cognitive impairment, most respondents stated that their relatives should make the decision for them.

Respondent: Look, when you're somewhat able to decide for yourself, I think you do this together with everyone involved. Well, and if that isn't an option, I have the impression that it's a decision that is made in agreement with the family and doctors. (…) But, in principle the decision is mine.

A minority of respondents thought that the physician should decide about starting CDS, as medical expertise was considered to be fundamental.

Respondent: So, his [the doctor's] medical knowledge is always decisive. And to be fair, when I think it's time, and he doesn't, well we have to discuss this because I don't want to overrule his medical knowledge. But yes, if you ask me explicitly, I think the doctor should make the assessment. Whether providing it [CDS] is rational.

The timing of conversations about CDS was also important. Respondents with experience as a relative were generally positive about early discussions on CDS, as this provided them with clear information and provided a sense of preparedness. However, in many cases, respondents said that CDS was discussed when a situation of refractory suffering was already at hand, and that it had not been a topic of conversation before that moment. According to some, conversations on death and treatment options in the dying phase were avoided in its entirety by both patients and HCPs. In these cases, the suggestion of CDS by the treating HCP sometimes came as a surprise.

Respondent: In the end, we weren't included in the discussion about her treatment. At a certain point, several persons who didn't know me or my mother entered the room and injected a sedative into her. To me, this was all very disrespectful. Because this is her… well her last. and this was not specified. They never clearly discussed her dying phase with us.

Interviewer: So you weren't included in the decision‐making process?

Respondent: No, while I was aware of what was happening due to my own knowledge. But I wasn't involved, no.

### 3.3 Experiences with the provision of CDS care

All respondents mentioned the importance of adequate communication and clear information by the involved HCPs. Several respondents with experience as a relative said that inadequate information provision and communication on CDS led to distressing situations. However, when expectations were managed by the HCP and patients and families were well informed on CDS care and potential complications—such as waking up—less distress was experienced.

Respondent: Well, I didn't know what it [CDS] entailed and neither did my father. My father said: ‘The doctor will be here soon, shall I lie down on the couch downstairs? In that way they don't have to carry me down the stairs when I'm gone’. But eventually, it took three days before he died. He just imagined it [CDS] to be something else than it was in reality. Well, the doctor administered the injections, and the home care nurses were supposed to ensure the medication would be repeated in time. But he woke up – which shouldn't have happened – and my father thought that he was gone but he wasn't. I thought that was horrible. To me, this was, very, very awful.

Taking time to connect with the patient and relatives, listening carefully and being receptive towards their input were considered to be essential aspects of communication by HCPs. Additionally, it was considered important that the HCP ensured that both relatives and patients understood the situation and were addressed in an appropriate manner, without the use of medical jargon.

Respondent: Well, that was a good conversation. She was accompanied by a physician in training. And my husband asked for careful explanation because he thought it resembled euthanasia. No, it is not euthanasia, it is helping with the dying process, and she would explain it a hundred times to him.

Most respondents who experienced CDS as a relative said that closely involved and available HCPs were of paramount importance to both the patient and themselves. In particular, mutual trust and understanding were important qualities in the relationship with the HCPs. Therefore, patients and relatives mostly preferred that their treating physician, with whom such a relationship was already established, provided CDS care.

Respondent: And when she was in a very poor condition, her physician went on a holiday for a week before she died. Well, we didn't like that, because we had a very good relationship with this man, and he was also the one she confided in. And on Wednesday another physician came to see her, and he said: well, we can start the palliative sedation. We can give you the sedation now. At that point, she already had morphine and such. But she didn't want that at all, because, well, she wanted to wait until her own physician returned from holiday.

Several respondents experienced that continuity of care was compromised when care had to be transferred from one provider to another and when staffing levels were low, for example outside regular working hours. Relatives repeatedly had to ask for care, as this was not timely provided in their view.

Respondent: But in the weekend… yes that's horrible. When you're in labor during weekends, everything carries on, but when you die you must wait until Monday.

### 3.4 Quality of dying with CDS

In almost all cases reported by the respondents, the patient died within one week after starting CDS. Respondents were largely satisfied with the quality of dying of their relatives under CDS; ‘a relief’ was frequently the word used to describe what they had experienced when CDS had commenced. The main reason for this was that CDS allowed the patient to die calmly, without any pain, restlessness or other suffering. Respondents often compared the dying of their relative to sleeping, which was considered comforting, and they were also appreciative of the idea of a gradual dying process during which the patient gently slides away into death.

Respondent: The whole night she just slept very well, and that last part was so good. You just see that she doesn't have to suffer anymore and that she was asleep, and was also not gone at once.

In various reported cases the patient showed signs of restlessness, which was considered to be undesirable. Incidentally, the patients woke up from sedation, and this was appreciated with mixed emotions by our respondents. For some, it was not upsetting, as they were aware that this could occur. However, others were very distressed when it happened.

Respondent: She moved her head restlessly from side to side, and she made fists with her hands. And her one leg moved restlessly. And her right hand was paralyzed, so we put a piece of cloth in her hand so she wouldn't hurt herself with her nails. Those kinds of things. She was just too agitated. For me, this was very difficult.

The fact that CDS implies loss of the patient's ability to communicate was not considered problematic by the respondents. Comfort for themselves or their relative was more important. However, when relatives were not counseled properly that communication is not possible after commencing sedation, this was a source of distress.

### 3.5 Distinction between euthanasia and CDS

In multiple cases, relatives reported experiences of hastening the patient's death by CDS. In some cases, this was explicitly discussed with the attending HCP, and in other instances, this was the perception of the relative or of the patient themselves. Hastening death was mostly considered a desirable effect of CDS in light of the patient's terminal condition.

Respondent: So, my husband woke up when the doctor prepared the sedative. And my brother‐in‐law and I said goodbye to him. And then the medication was administered, but nothing happened. He just stayed alive. And the doctor thought that he would have died while administering the morphine. But that didn't happen. He [the doctor] said: sometimes that happens. And then he gave him the sedative. And my husband still didn't die. And then our doctor said: well, I don't know how he does it, but he's still alive.

When discussing their views on palliative care for themselves, many respondents held the opinion that it was a matter of choice or preference whether euthanasia or CDS should be used to relieve their suffering. Respondents who preferred CDS over euthanasia mentioned that they appreciated CDS as this is a more gradual process allowing them to calmly die without needless suffering. They also thought that CDS would be more acceptable to relatives, and less difficult for physicians compared to euthanasia. Additionally, multiple respondents indicated that CDS is acceptable from a religious standpoint. Respondents who preferred euthanasia over CDS brought up that euthanasia accommodates more personal agency and avoids a potentially long and burdensome terminal phase.

Several respondents indicated that, although in general they preferred euthanasia, in certain circumstances CDS would be preferential to them. Mainly when the procedure towards euthanasia would be too time‐consuming, for example, when suffering was a result of an acute situation, or when cognitive problems would make euthanasia impossible. These were also important reasons in several of the discussed cases to revert from euthanasia to CDS. In most of these cases, the clinical situation deteriorated rapidly, leaving no time to start up the euthanasia protocol. In other cases, euthanasia was no option due to a lack of competence on the part of the patient, for example, due to stroke or dementia.

### 3.6 Perceptions of CDS

Most respondents were aware of the main principles of CDS. However, some of the respondents did not display a correct understanding of CDS before clarification by means of a teach‐back. For example, several respondents thought that CDS comprised pain control without necessarily lowering the patient's consciousness. In a few cases, CDS was confused with starvation in the absence of lowering consciousness. In paricular, respondents who were included as patients and who did not have lived experience with CDS as relatives misunderstood the concept of CDS.

Almost all respondents were aware that palliative sedation is distinguished from active life termination, but many believed that palliative sedation hastens death, for example by means of starvation or highly dosed medication.

The respondents' initial perceptions of CDS were informed through various sources, such as newspaper articles and the internet, but also through personal contacts, earlier experience with CDS and discussions with HCPs.

## 4 DISCUSSION

Relatives were generally positive about their experience with CDS, especially when their loved‐one died peacefully. Situations of unbearable suffering during the dying phase were considered unacceptable by patients and relatives, and a calm and peaceful death was seen as crucial. The suffering of a dying patient called for intervention leading to the initiation of CDS. The reported suffering of patients was mostly caused by pain, restlessness, and dyspnea. However, in several cases, existential suffering or the prevention of suffering was mentioned as the main motivation to start CDS. This potential broadening of the indication is perhaps one reason for the increased practice of CDS in end‐of‐life care.

In our interviews CDS was often thought of as a matter of choice by the patients and families, in which the patient decides and the physician serves as an advisor, reflecting the importance of self‐agency at the end stage of life. Distress often arose from a lack of feeling in control, and especially a lack of involvement in decision‐making on CDS was a major concern for relatives. Tensions related to communication and involvement may be caused by divergent views on responsibility and decision‐making about CDS among patients, relatives and HCPs. On the one hand, CDS is traditionally regarded as a ‘last resort’ medical decision, for which a physician is ultimately responsible.^2^, ^3^, ^4^, ^6^ On the other hand, there is strong agreement that the key to improvement of end‐of‐life care is to make the care consistent with patient preferences by an individualized process of decision‐making.^26^, ^27^ In our study, respondents often said that the decision was eventually made by the patient or relative, the latter in the case of cognitive impairment of the patient. Many saw the role of physicians mainly as advising on available end‐of‐life care options, and on the right timing for CDS initiation. These results differ from similar research conducted ten years ago when relatives reported that the final decision was made by the attending physician.^21^ Nevertheless, in recent years research has shown that HCPs put more emphasis on the wishes of patients and relatives.^16^, ^17^, ^19^ A study involving HCPs from the United Kingdom, Belgium and The Netherlands showed that the Belgian HCPs tend to frame CDS as a regular end‐of‐life care option for which the patient can choose.^28^ The dominant view among our respondents of CDS as a normal palliative care option for which they can choose instead of a last resort informed by a medical judgment on the refractory state of symptoms may contribute to an increase in requests for CDS.

The wish for a calm and peaceful death was so important that moral problems with CDS raised in the literature were of no concern to our respondents. The difference between CDS and euthanasia was recognized, but still, most respondents thought that CDS potentially hastens death—which is usually considered key to the ethical distinction between CDS and euthanasia.^2^, ^3^, ^4^ However, the idea of respondents that CDS potentially hastens death was actually viewed as acceptable by them, as death was a better alternative than unbearable suffering. This relates to another ethical concern regarding the distinction between CDS and euthanasia. Namely, it has been suggested that CDS results in the social death of the patient due to loss of awareness and thus communication.^9^ However, losing the ability to communicate was mostly not experienced as problematic by the relatives in our study.

In general, our respondents were satisfied with the quality of CDS and the care they received. We identified several determinants of good quality of death with CDS. First and foremost, respondents appreciated CDS when it allowed the patient to die a calm and peaceful death. It was considered ‘a relieve’ when the suffering of their loved one had ended due to CDS. The gradual nature of CDS, in which the patient slides away into death while seemingly asleep, was considered comforting for both patient and relative and added to a positive experience of CDS. This was often contrasted with euthanasia, which some thought to be too abrupt. Respondents valued it when continuity of care was guaranteed and when CDS was attended by their own physician. Many of our respondents reported that CDS was appropriately discussed by HCPs, which was appreciated as it enhanced understanding and managed expectations of CDS. However, for several respondents, CDS was also a source of distress. Unmet expectations, inadequate communication and information provision, and difficulties in understanding CDS contributed to the distress and reduced the experienced quality of CDS. Adverse experiences regarding communication and information provision were also reported in other studies.^21^, ^23^, ^29^, ^30^ This underlines the importance of timely and adequate communication on end‐of‐life decisions including CDS with both patients and relatives.

Most respondents were able to give an accurate description of palliative sedation and CDS, and were informed through media exposure, earlier experiences of end‐of‐life care, and advance care discussion with HCPs. Improved attention on end‐of‐life care in the public may partly explain the increase in CDS, as patients and relatives are better aware of palliative care options. However, some respondents misunderstood CDS: starvation, pain reduction and abstaining from life‐prolonging measures in the absence of lowering a patient's consciousness were also considered to be palliative sedation by some. This finding corresponds with earlier research among the general public in The Netherlands, in which the term palliative sedation was also indistinct.^31^ The misunderstanding was most prominent in the respondent group without lived experience of CDS as a relative. The group that experienced CDS as a relative, was mostly aware of the important principles of CDS.

When situating our results within the evolving practice of CDS, several explanations from the perspective of patients and relatives for the increase of CDS can be suggested. First, there seems to be a shift in indication assessment, as experienced patients and relatives sometimes report that CDS is currently used to relieve existential suffering. Second, patients and relatives emphasize the importance of comfort at the end‐stage of life, and desire agency over the decision‐making on palliative care options in this phase. Lastly, CDS may be requested more often as respondents were better informed on end‐of‐life care.

### 4.1 Strengths and limitations

The main strength of this study is that the in‐depth interviews allowed uncensored insight into the experiences and perceptions of CDS of both patients and relatives within an evolving practice of CDS. However, several limitations may have influenced our results. Most importantly, some respondents—especially those without lived experience with relatives—initially misunderstood the term CDS/palliative sedation, although we corrected this in our interviews and excluded respondents who did not receive a teach‐back it could be that this influenced our results. Secondly, our respondents were mainly highly educated Caucasian patients at a tertiary care center in the middle of The Netherlands. These respondents are more likely to emphasize the importance of open communication and good quality of dying. Whereas it is known that people from non‐Western cultures often have different ideas on good palliative care.^32^, ^33^ Unfortunately, we were not able to include respondents with non‐Western cultural backgrounds. This raises questions on the generalizability to non‐Western populations as their perceptions of CDS are probably not reflected in this study. Thirdly, potential respondents were not selected randomly, and therefore selection bias is possible, especially respondents with an interest in end‐of‐life may be more likely to apply for participation. Lastly, recall bias may have played a role, as the first case occurred in 2009.

## 5 CONCLUSION

The traditional view of CDS as a last resort option for a physician to relieve a patient's suffering at the end of life is not present among patients and relatives in our study. Instead, our results show that they perceive CDS as a regular—and not an exceptional—palliative care option. Along with this normalization of CDS, patients and relatives claim a substantial say in the decision‐making and are mainly motivated by a wish to avoid suffering and exercise control at the end of life. This may result in an increase in CDS requests. The distinct views on CDS should be reconciled in guidelines and protocols for CDS. This can be done by introducing a shared‐decision model in which the HCP, the patient and relatives are responsible for deciding on CDS, and not primarily the physician. By doing so, guidelines will better reflect the current practice of CDS.

## AUTHOR CONTRIBUTIONS

Judith A. C. Rietjens, Agnes van der Heide, Johannes J. M. van Delden, Ghislaine J. M. W. van Thiel and Geeske. Hendriksen conceived and designed the project, Louise Annemoon Jonker and Madelon. T. Heijltjes conducted the interviews and Ghislaine J. M. W. van Thiel supervised the conduction of the interviews, Lousie Annemoon Jonker, Madelon, T. Heijltjes and Ghislaine J. M. W. van Thiel analyzed the data, Louise Annemoon Jonker took the lead in writing the manuscript and all authors discussed the results during thematic discussions and contributed to the final manuscript.

## CONFLICT OF INTEREST STATEMENT

The authors declare no conflict of interest.

## Supporting information

Supporting information.

## Data Availability

The authors confirm that the data supporting the findings of this study are available within the manuscript and supplemental files. Raw data that support the findings of this study are available from the corresponding author, upon reasonable request.

## References

[1] Graeff AD , Dean M . Palliative sedation therapy in the last weeks of life: a literature review and recommendations for standards. J Palliat Med. 2007;10(1):67‐85. 10.1089/jpm.2006.0139 17298256

[2] Royal Dutch Medical Association (KNMG) . Guideline Palliative Sedation. 3rd ed. KNMG.

[3] Royal Dutch Medical Association (KNMG) . Guideline Palliative Sedation. 2th ed. KNMG.

[4] Royal Dutch Medical Association (KNMG) . Guideline Palliative Sedation. 1st ed. KNMG.

[5] Maltoni M , Scarpi E , Rosati M , et al. Palliative sedation in end‐of‐life care and survival: a systematic review. J Clin Oncol. 2012;30(12):1378‐1383. 10.1200/JCO.2011.37.3795 22412129

[6] Abarshi E , Rietjens J , Robijn L , et al. International variations in clinical practice guidelines for palliative sedation: a systematic review. BMJ Support Palliat Care. 2017;7(3):223‐229. 10.1136/bmjspcare-2016-001159 28432090

[7] Rietjens JAC , Heijltjes MT , van Delden JJM , Onwuteaka‐Philipsen BD , van der Heide A . The rising frequency of continuous deep sedation in the Netherlands, a repeated Cross‐Sectional Survey in 2005, 2010, and 2015. J Am Med Dir Assoc. 2019;20(11):1367‐1372. 10.1016/j.jamda.2019.06.012 31378702

[8] Booker R , Bruce A . Palliative sedation and medical assistance in dying: distinctly different or simply semantics? Nurs Inquiry. 2020;27(1):e12321. 10.1111/nin.12321 PMC9285680 31756038

[9] Janssens R , van Delden JJM , Widdershoven GAM . Palliative sedation: not just normal medical practice. Ethical reflections on the Royal Dutch Medical Association's guideline on palliative sedation. J Med Ethics. 2012;38(11):664‐668. 10.1136/medethics-2011-100353 22811556

[10] Materstvedt LJ , Bosshard G . Deep and continuous palliative sedation (terminal sedation): clinical‐ethical and philosophical aspects. Lancet Oncol. 2009;10(6):622‐627. 10.1016/S1470-2045(09)70032-4 19482251

[11] Rietjens JA , van Delden JJ , van der Heide A . Palliative sedation: the end of heated debate? Palliat Med. 2018;32(11):1639‐1640. 10.1177/0269216318762708 29532705

[12] Benítez‐Rosario MA , Ascanio‐León B . Palliative sedation: beliefs and decision‐making among Spanish palliative care physicians. Supp Care Cancer. 2020;28(6):2651‐2658. 10.1007/s00520-019-05086-4 31637516

[13] Muishout G , van Laarhoven HWM , Wiegers G , Popp‐Baier U . Muslim physicians and palliative care: attitudes towards the use of palliative sedation. Supp Care Cancer. 2018;26(11):3701‐3710. 10.1007/s00520-018-4229-7 PMC6182360 29736869

[14] Lokker ME , Swart SJ , Rietjens JAC , van Zuylen L , Perez RSGM , van der Heide A . Palliative sedation and moral distress: a qualitative study of nurses. Appl Nurs Res. 2018;40:157‐161. 10.1016/j.apnr.2018.02.002 29579492

[15] Heijltjes MT , Morita T , Mori M , et al. Physicians' opinion and practice with the continuous use of sedatives in the last days of life. J Pain Symptom Manage. 2022;63(1):78‐87. 10.1016/j.jpainsymman.2021.07.012 34333097

[16] Robijn L , Chambaere K , Raus K , Rietjens J , Deliens L . Reasons for continuous sedation until death in cancer patients: a qualitative interview study. Eur J Cancer Care. 2017;26(1):e12405. 10.1111/ecc.12405 26515814

[17] Raus K , Brown J , Seale C , et al. Continuous sedation until death: the everyday moral reasoning of physicians, nurses and family caregivers in the UK, The Netherlands and Belgium. BMC Med Ethics. 2014;15:14. 10.1186/1472-6939-15-14 24555871 PMC3942295

[18] Detering KM , Hancock AD , Reade MC , Silvester W . The impact of advance care planning on end of life care in elderly patients: randomised controlled trial. BMJ. 2010;340:c1345. 10.1136/bmj.c1345 20332506 PMC2844949

[19] Kuosmanen L , Hupli M , Ahtiluoto S , Haavisto E . Patient participation in shared decision‐making in palliative care—an integrative review. J Clin Nurs. 2021;30(23‐24):3415‐3428. 10.1111/jocn.15866 34028923

[20] Bruinsma S , Rietjens J , van der Heide A . Palliative sedation: a focus group study on the experiences of relatives. J Palliat Med. 2013;16(4):349‐355. 10.1089/jpm.2012.0410 23421537

[21] Bruinsma SM , Rietjens JAC , Seymour JE , Anquinet L , van der Heide A . The experiences of relatives with the practice of palliative sedation: a systematic review. J Pain Symptom Manage. 2012;44(3):431‐445. 10.1016/j.jpainsymman.2011.09.006 22658470

[22] Morita T , Ikenaga M , Adachi I , et al. Family experience with palliative sedation therapy for terminally ill cancer patients. J Pain Symptom Manage. 2004;28(6):557‐565. 10.1016/j.jpainsymman.2004.03.004 15645586

[23] Tursunov O , Cherny N , Ganz F . Experiences of family members of dying patients receiving palliative sedation. Oncol Nurs Forum. 2016;43(6):E226‐E232. 10.1188/16.ONF.E226-E232 27768142

[24] van Dooren S , van Veluw HTM , van Zuylen L , Rietjens JAC , Passchier J , van der Rijt CCD . Exploration of concerns of relatives during continuous palliative sedation of their family members with cancer. J Pain Symptom Manage. 2009;38(3):452‐459. 10.1016/j.jpainsymman.2008.11.011 19559563

[25] Tong A , Sainsbury P , Craig J . Consolidated criteria for reporting qualitative research (COREQ): a 32‐item checklist for interviews and focus groups. Int J Qual Health Care. 2007;19(6):349‐357. 10.1093/intqhc/mzm042 17872937

[26] Ingravallo F , de Nooijer K , Pucci V , et al. Discussions about palliative sedation in hospice: frequency, timing and factors associated with patient involvement. Eur J Cancer Care. 2019;28(3):e13019. 10.1111/ecc.13019 30773765

[27] Houska A , Loučka M . Patients' autonomy at the end of life: a critical review. J Pain Symptom Manage. 2019;57(4):835‐845. 10.1016/j.jpainsymman.2018.12.339 30611709

[28] Seymour J , Rietjens J , Bruinsma S , et al. Using continuous sedation until death for cancer patients: a qualitative interview study of physicians' and nurses' practice in three European countries. Palliat Med. 2015;29(1):48‐59. 10.1177/0269216314543319 25062816 PMC4266692

[29] Eun Y , Hong IW , Bruera E , Kang JH . Qualitative study on the perceptions of terminally ill cancer patients and their family members regarding end‐of‐life experiences focusing on palliative sedation. J Pain Symptom Manage. 2017;53(6):1010‐1016. 10.1016/j.jpainsymman.2016.12.353 28192224

[30] Virdun C , Luckett T , Davidson PM , Phillips J . Dying in the hospital setting: a systematic review of quantitative studies identifying the elements of end‐of‐life care that patients and their families rank as being most important. Palliat Med. 2015;29(9):774‐796. 10.1177/0269216315583032 25921707 PMC4572388

[31] van der Kallen HT , Raijmakers NJ , Rietjens JA , et al. Opinions of the Dutch public on palliative sedation: a mixed‐methods approach. Br J Gen Pract. 2013;63(615):e676‐e682. 10.3399/bjgp13X673685 24152482 PMC3782800

[32] de Graaff FM , Mistiaen P , Devillé WL , Francke AL . Perspectives on care and communication involving incurably ill Turkish and Moroccan patients, relatives and professionals: a systematic literature review. BMC Palliat Care. 2012;11:17. 10.1186/1472-684X-11-17 22985103 PMC3517329

[33] de Graaff FM , Francke AL , van den Muijsenbergh ME , van der Geest S. “Palliative care”: A contradiction in terms? A qualitative study of cancer patients with a Turkish or Moroccan background, their relatives and care providers. BMC Palliat Care. 2010;9:19. 10.1186/1472-684X-9-19 20831777 PMC2944252

