# Supplementary material for: Experiences and perceptions of continuous deep sedation: An interview study among Dutch patients and relatives
Source: Health Expect. 2023 Oct 11;27(1):e13869. doi: 10.1111/hex.13869 (PMC10726059; doi:10.1111/hex.13869)
Supplement: Supplementary file 1 — Supporting information. [file HEX-27-e13869-s001.docx]

| **Interview guide, semi-structured interview with relatives** | |
| --- | --- |
| Introduction interview palliative sedation  Today we are going to discuss the topic palliative sedation, as you experienced this up close with your relative.  When reflecting on the dying of your relative  Your relative passed away some time ago. Can you tell how his or her last days were? | |
| Terugkijkend op het overlijden van naaste, gestructureerd | |
| *Casus* | - **What was the reason to start considering palliative sedation?** - **When was palliative sedation discussed for the first time?**   *Who initiated this conversation?*   - **Which healthcare professionals were involved, and at what time?**   *How was the communication with them?*   - **How did the decision-making process go?**   *What were the most important reasons for the decision?*  *Did your relative feel involved in the decision?*  *How were you as a relative involved in the decision?*   - **How did the palliative sedation go?**   *Did your relative wake up from the sedation at any point?*  *What did you think about the performance of the medical staff?*   - **Did you know how the palliative sedation was performed?**   *Pumps and equipment*  *Medication*  *Fluids and nutrition*   - **Was euthanasia an option, and how was this discussed?** - **What were your expectations of palliative sedation?** - **How do you look back on the palliative sedation and the passing of your relative?**   *Was it difficult for you that you could not communicate with your relative?* |
| Own perceptions | |
| *Passing of relative* | How would you describe the passing of your relative in a few words? |
| *Dying* | Did the passing of your relative influence your own ideas of dying? |
| *Palliative sedation, own perception* | What do you think about giving CDS to someone who is terminally ill? Do you think palliative sedation should be applied when someone suffers unbearably from psychological symptoms at the end-stage of life? |
| *Euthanasia vs palliative sedation* | Do you thinks euthanasia differs from CDS? Why ? |
| *Stands:*  My relative and me felt adequately informed on palliative sedation  My relative suffered unbearably before palliative sedation commenced  I think palliative sedation made dying more pleasant for my relative. | |
|  | |

| **Interview guide, semi-structured interview with patients.** | |
| --- | --- |
| Introduction interview palliative sedation  Today we would like to discuss palliative sedation because you have considered it, or are considering this for yourself. | |
| When reflecting on your thougts | |
| *Casus* | - **Can you tell what the reason was to start considering CDS?** - **What is your expectation of CDS?**   *What would palliative sedation yield?*  *How do you know when it is ‘time’ for palliative sedation, and who would indicate this?*   - **What do you like about palliative sedation, and what don’t you like?** - **Have you discussed palliative sedation with others?**   *Relavies? 🡪 who started this conversation*  *Healthcare professionals? 🡪 Who initiated this conversation?*  *🡪 How is the relationship with them?*  *🡪 Are they easiliy accessible?*   - **What are important elements in the decision for palliative sedation?**   *Does the loss of ability to communicate with your relatives play a role in your decision?*   - **Have you experienced palliative sedation with someone else before?**   *Did this change your view on dying?*   - ***Do you know how palliative sedation is performed?***   *How did you receive this knowledge?*   - **What do you think about euthanasia?**   *Is euthanasia an option for you?*  *Is euthanasia different from palliative sedation?*   - **Would you consider palliative sedation if you suffer unbearably from psychological symptoms in the last days of your life?** |
|  | |
|  | |
